# Supplementary material for: Machine Learning Applied to Pre-Operative Computed-Tomography-Based Radiomic Features Can Accurately Differentiate Uterine Leiomyoma from Leiomyosarcoma: A Pilot Study
Source: Cancers (Basel). 2024 Apr 19;16(8):1570. doi: 10.3390/cancers16081570 (PMC11048510; doi:10.3390/cancers16081570)
Supplement: Supplementary file 1 [file cancers-16-01570-s001.zip › cancers-2920716-supplementary.pdf]

Supplementary tables & figures

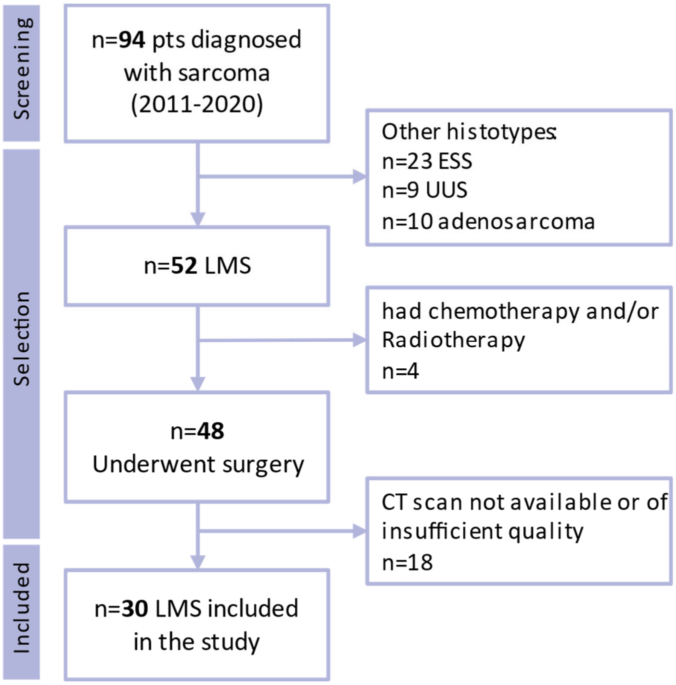

Supplementary Figure S1. *Selection of patients with LMS included in this study. LMS: leiomyosarcoma; ESS: endometrial stromal sarcoma; UUS: undifferentiated uterine sarcoma; CT: computed tomography, n: number; pts: patients.*

Supplementary Table S1. *Demographic characteristics and clinical parameters of the patients diagnosed with uterine leiomyosarcomas and myomas. N: number; LMS: leiomyosarcoma; UM: myoma; BMI: Body Mass Index; SD: standard deviation.*

	**Patient group**		**p-value**
**Variable**	**LMS N=30**	**UM N=35**	
**Age (years)** Mean±SD	59.6±10.1	48.5±11.2	<0.001
**BMI (kg/m^2)** Mean±SD	26.6±5.7	23.8±4.3	0.03
**Menopause status at diagnosis** N(%)	27(23.8%)	15(42.9%)	<0.001
**Hormonal therapy** N(%)	6(20%)	11(31.4%)	0.45

Supplementary Table S2. *Clinical and oncological parameters of the patients diagnosed with uterine leiomyosarcoma. *Patients with two recurrent sites were as follows: one patient with lung and peritoneum; one patient with aortic lymph nodes and lung; one patient with thoracic and abdominal recurrence. LMS: leiomyosarcoma.*

**Variable**	**LMS N=30**		**UM N=35**	
**Pathological subtypes** N(%)	**Conventional spindle cell**	25(83.3%)	**Typical**	38(74.5%)
	**Epithelioid**	3(10%)	**Degenerated**	10(19.6%)
	**Myxoid**	2(6.7%)	**Cellulated**	3(5.9%)
**Stage (FIGO 2014)** N(%)	**I**	23(76.7%)		
	**II**	3(10%)		
	**III**	1(3.3%)		
	**IV**	3(10%)		
**Adjuvant treatment** N(%)		22(66.7%)		
**Recurrence status** N(%)	**No**	6(20%)		
	**Yes**	21(70%)		
	**Persistent disease**	3(10%)		
**Recurrence site** N(%)	**Vaginal**	1(5%)		
	**Pelvic**	3(15%)		
	**Pulmonary**	9(45%)		
	**Liver**	1(5%)		
	**Bone**	1(5%)		
	**Abdomen**	1(5%)		
	**Two sites***	3(15%)		
**Recurrence treatment** N(%)	**Surgery**	1(4.8%)		
	**Chemotherapy**	9(42.9%)		
	**Surgery + Chemotherapy**	3(14.3%)		
	**Chemotherapy + Radiotherapy**	3(14.3%)		
	**Surgery + Chemotherapy + Radiotherapy**	1(4.8%)		
	**Palliative treatment**	3(14.3%)		
	**None**	1(4.8%)		

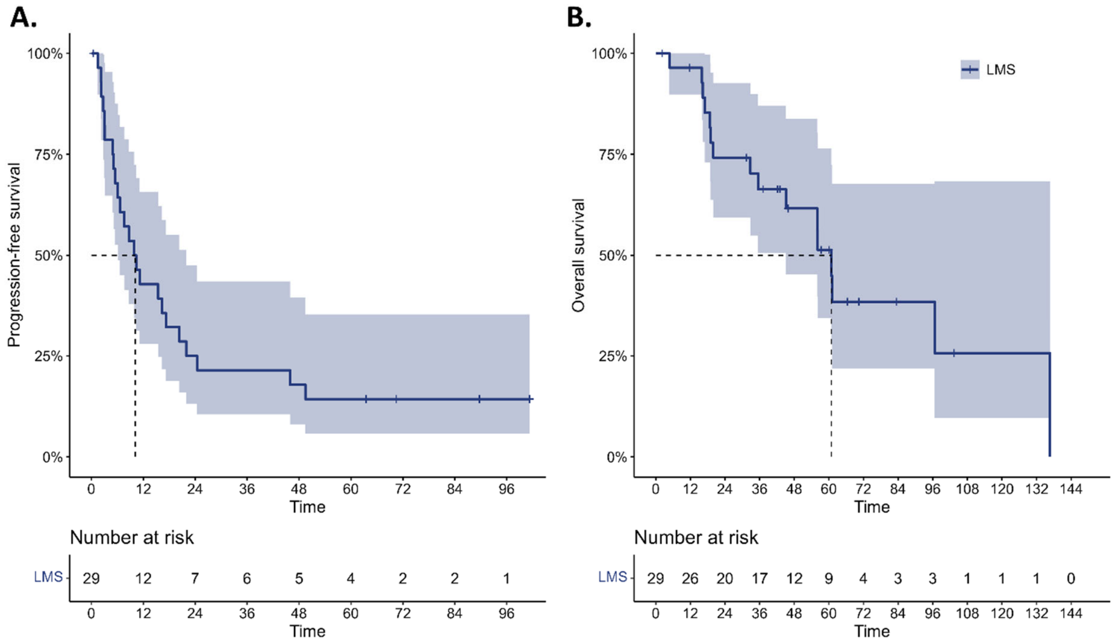

Supplementary Figure S2. *Kaplan-Meier curves with 95% CI of patients diagnosed with leiomyosarcoma showing A. Progression-free survival; B. Overall survival. CI: confidence interval.*

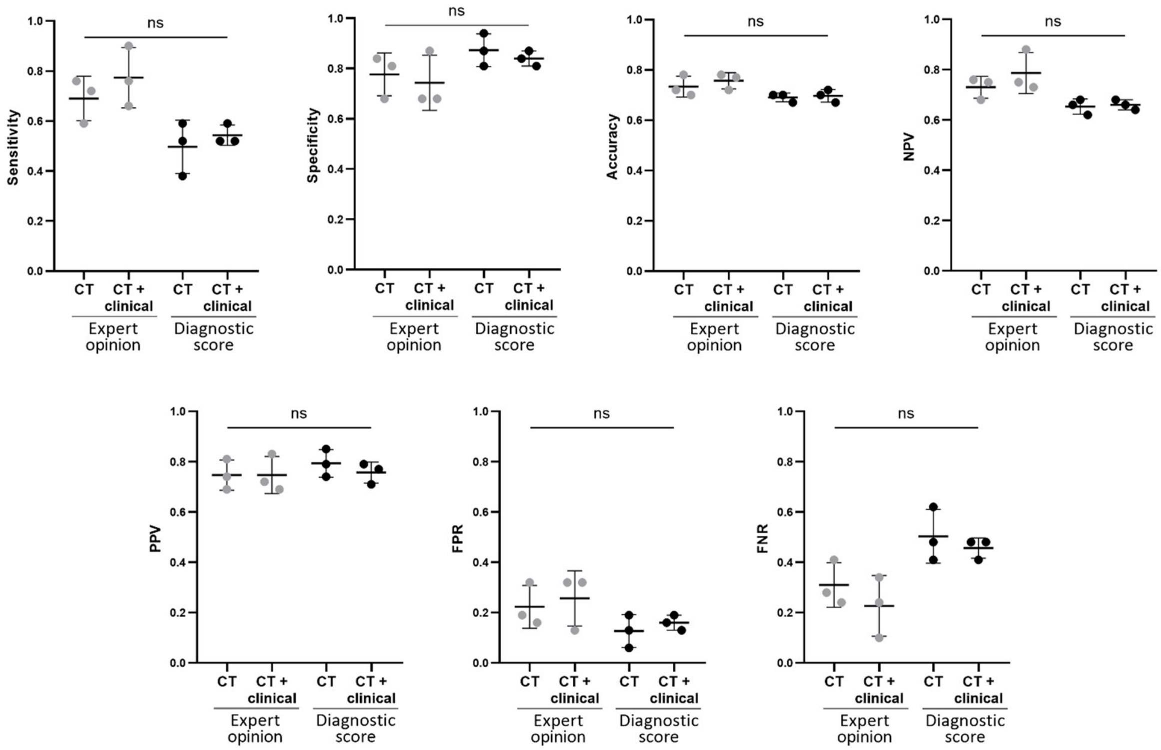

Supplementary Figure S3. *ROC parameters of differential diagnosis capacity of expert radiologists. Comparison between ROC parameters obtained by radiologists when evaluating the CT scans alone vs with the clinical file of the patients and the results obtained with the scoring system. CT: computer tomography; MD: medical doctor (evaluator-radiologist); ns: p-value not significant; NPV: negative predictive value; PPV: positive predictive value; FPR: false positive rate; FNR: false negative rate.*

Supplementary Table S3. Performance of classifier models according to the Feature selection model and radiomic feature class-group.

Feature selection model	Input Variables	+ GLM						+ RF						+ SVM					
		Training			Test			Training			Test			Training			Test		
		AUC (95%CI)	Se	Sp	AUC (95%CI)	Se	Sp	AUC (95%CI)	Se	Sp	AUC (95%CI)	Se	Sp	AUC (95%CI)	Se	Sp	AUC (95%CI)	Se	Sp
**LASSO**	**All In**	0.94 (0.88-0.99)	0.86	0.92	0.82 (0.65-0.99)	0.78	0.87	1.00 (1.00-1.00)	1.00	1.00	0.97 (0.90-1.00)	1.00	0.93	0.93 (0.87-1.00)	0.95	0.92	0.69 (0.50-0.87)	0.44	0.93
**Boruta**	**Only First order**	NA	NA	NA	NA	NA	NA	1.00 (1.00-1.00)	1.00	1.00	0.88 (0.73-1.00)	0.89	0.97	0.81 (0.70-0.92)	0.67	0.94	0.69 (0.51-0.87)	0.44	0.93
**Boruta**	**Only Texture**	0.91 (0.82-0.99)	0.81	0.94	0.78 (0.62-0.94)	0.89	0.67	1.00 (1.00-1.00)	1.00	1.00	0.97 (0.90-1.00)	1.00	0.93	1.00 (1.00-1.00)	1.00	1.00	0.74 (0.56-0.93)	0.56	0.93
**Boruta**	**All In**	0.91 (0.82-0.99)	0.81	0.94	0.78 (0.62-0.94)	0.89	0.67	1.00 (1.00-1.00)	1.00	1.00	0.97 (0.90-1.00)	1.00	0.93	1.00 (1.00-1.00)	1.00	1.00	0.80 (0.62-0.98)	0.67	0.93
**RFE**	**Only First order**	NA	NA	NA	NA	NA	NA	1.00 (1.00-1.00)	1.00	1.00	0.88 (0.74-1.00)	0.89	0.87	0.81 (0.70-0.92)	0.67	0.94	0.69 (0.51-0.87)	0.44	0.93
**RFE**	**Only Texture**	0.92 (0.84-1.00)	0.86	0.89	0.81 (0.65-0.97)	0.89	0.73	1.00 (1.00-1.00)	1.00	1.00	0.94 (0.84-1.00)	0.89	1.00	1.00 (1.00-1.00)	1.00	1.00	0.71 (0.52-0.90)	0.56	0.87
**RFE**	**All In**	0.92 (0.84-1.00)	0.86	0.89	0.81 (0.65-0.97)	0.89	0.73	1.00 (1.00-1.00)	1.00	1.00	0.97 (0.90-1.00)	1.00	0.93	1.00 (1.00-1.00)	1.00	1.00	0.74 (0.58-0.91)	0.89	0.60
